# The relevance of the unique anatomy of the human prefrontal operculum to the emergence of speech

**DOI:** 10.1038/s42003-023-05066-9

**Published:** 2023-07-05

**Authors:** Céline Amiez, Charles Verstraete, Jérôme Sallet, Fadila Hadj-Bouziane, Suliann Ben Hamed, Adrien Meguerditchian, Emmanuel Procyk, Charles R. E. Wilson, Michael Petrides, Chet C. Sherwood, William D. Hopkins

**Affiliations:** 1 Univ Lyon, Université Lyon 1, Inserm, Stem Cell and Brain Research Institute, U1208 Bron, France; 2 Institut du Cerveau et de la Moelle épinière, Sorbonne Université, Inserm, CNRS, Paris, France; 3 Wellcome Integrative Neuroimaging Centre, Department of Experimental Psychology, University of Oxford, Oxford, OX1 3SR UK; 4 Integrative Multisensory Perception Action & Cognition Team (ImpAct), INSERM U1028, CNRS UMR5292, Lyon Neuroscience Research Center (CRNL), Lyon, France; 5 University of Lyon 1, Lyon, France; 6 Institut des Sciences Cognitives Marc Jeannerod, UMR5229, CNRS-Université Claude Bernard Lyon I, Bron, France; 7 Laboratoire de Psychologie Cognitive, UMR7290, Aix-Marseille Université, CNRS, 13331 Marseille, France; 8 Station de Primatologie CNRS, UAR846, 13790 Rousset, France; 9 Institut Language, Communication and the Brain (ILCB), Aix-Marseille Université, 13604 Aix-en-Provence, France; 10 Montreal Neurological Institute, Department of Neurology and Neurosurgery and Department of Psychology, McGill University, Montreal, Quebec Canada; 11 Department of Anthropology and Center for the Advanced Study of Human Paleobiology, The George Washington University, Washington, DC USA; 12 Department of Comparative Medicine, University of Texas MD Anderson Cancer Center, Bastrop, Texas USA

**Keywords:** Neuroscience, Evolution

## Abstract

Identifying the evolutionary origins of human speech remains a topic of intense scientific interest. Here we describe a unique feature of adult human neuroanatomy compared to chimpanzees and other primates that may provide an explanation of changes that occurred to enable the capacity for speech. That feature is the Prefrontal extent of the Frontal Operculum (PFOp) region, which is located in the ventrolateral prefrontal cortex, adjacent and ventromedial to the classical Broca’s area. We also show that, in chimpanzees, individuals with the most human-like PFOp, particularly in the left hemisphere, have greater oro-facial and vocal motor control abilities. This critical discovery, when combined with recent paleontological evidence, suggests that the PFOp is a recently evolved feature of human cortical structure (perhaps limited to the genus *Homo*) that emerged in response to increasing selection for cognitive and motor functions evident in modern speech abilities.

## Abstract (web summary)

Comparisons of fMRI data from humans and chimpanzees suggests that the Prefrontal extent of the Frontal Operculum region recently evolved in humans possibly in relation to the emergence of modern speech.

## Introduction

Speech and its cognitive and motor control are central to human social communication. Although the neural basis for speech has been the subject of intense research, its origins remain poorly understood. An early theory of the absence of speech in nonhuman primates suggested that the higher position of the larynx limits the vocal tract’s range of sound articulation, but this has been refuted^1^. It turned out that these constraints are not sufficient to prevent monkeys from producing vocalizations comparable to human vowels^1^. The inability to speak was therefore to be sought elsewhere, likely in the organization of their cognitive and cerebral systems. More recently, neurogenetic adaptations have been shown (e.g., *FOXP2*), but the acquisition of human-specific DNA nucleotide changes are difficult to relate to the onset of speech and language abilities in hominin ancestors^2^. With the development of comparative sulcal-based, connectivity-based and functional neuroimaging studies, there is a growing literature showing that most of the neural structure for speech in the frontal cortex is already present in nonhuman primates^3^, including a clear homolog to Broca’s area^3–6^. The question that therefore arises is how a uniquely human ability might have evolved from relatively subtle modifications to brain anatomical structures that are shared in common with other primate species.

By combining sulcal pattern analysis with resting-state functional magnetic resonance imaging and cytoarchitectonic analysis, we have recently shown that much of the frontal cortex, including the medial frontal, the posterior frontal, the dorsolateral, and the frontopolar cortex, displays the same general principles of organization in chimpanzee, baboon, macaque, and human brains^6,7^. However, the ventrolateral prefrontal cortex varies distinctly in its morphology among cercopithecid monkeys, great apes, and humans. Understanding ventrolateral prefrontal cortex variation in primates is critical to determining the neuroanatomical changes that underlie human-specific abilities such as the emergence of speech.

**Fig. 1:**
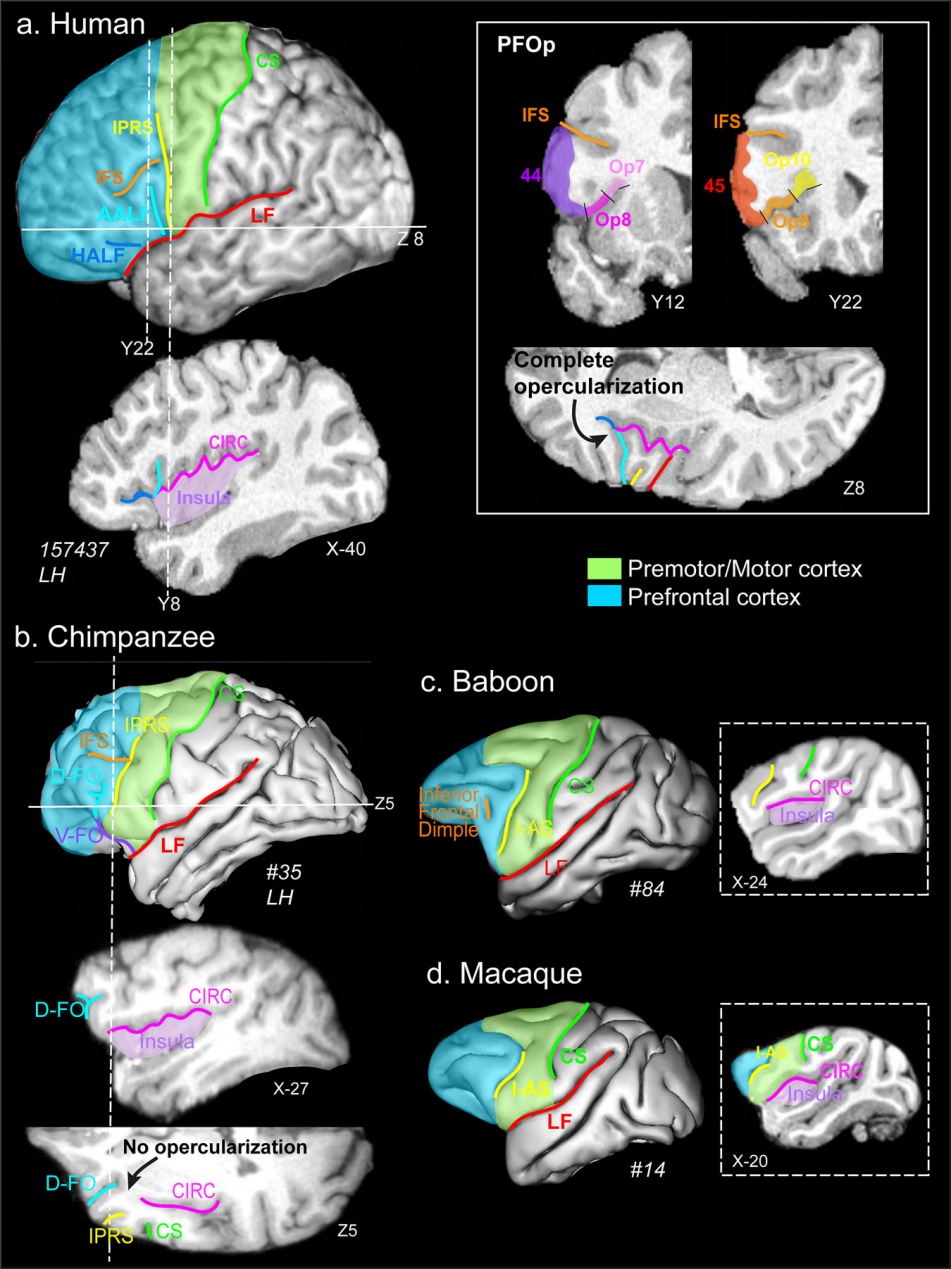
Morphological organization of the ventrolateral prefrontal cortex and its relationship with the insula in typical human, chimpanzee, baboon, and macaque(s) brains as shown on cortical surfaces, parasagittal, coronal, and axial sections of typical brains of each species. In all species, the premotor/motor cortex and prefrontal part of the frontal cortex are represented in green and blue areas. **a** **Humans.** A cortical surface of a typical human brain registered in the Montreal Neurological Institute (MNI) stereotaxic standard space shows that the ventrolateral prefrontal cortex is characterized by the presence of an inferior precentral sulcus (*IPRS*, in yellow), located just anterior to *CS* (green), *AALF* (light blue) and *HALF* (dark blue), *IFS* (orange), and *LF* (red). The parasagittal section shows that *CIRC* (and thus the insula) extends in the prefrontal cortex, i.e., rostral to *IPRS* (as displayed by the dotted line at MNI coordinate Y8). The two dotted lines on the cortical surface (left panel) display the anteroposterior level corresponding to the coronal sections displaying the four cytoarchitectonic areas lying in PFOp (i.e., Op7, Op8, Op9, Op10, right panel). The horizontal line on the cortical section shows the ventro-dorsal level of the axial section presented on the right panel. Results show that *AALF* joins CIRC, creating a complete opercularization, thus enlarging and creating a more comple PFOp. **b** Chimpanzees. A cortical surface of a typical chimpanzee brain registered in the chimpanzee standard space shows that the ventrolateral prefrontal cortex is characterized by the presence of an *IPRS* (yellow) just in front of *CS* (green), the *IFS* (in orange), and the dorsal -lateral- part of the *D-FO* (light blue), *V-FO* (purple) running on the orbitofrontal cortex. The dotted lines on the cortical surface displays the antero-posterior and dorso-ventral levels of, respectively, the parasagittal and axial sections presented on the right panel. Data show that the rostralmost part of *CIRC* reaches the level of *IPRS* without reaching *D-FO* (the homolog of the human AALF), thus preventing a full opercularization. **c**, **d** Baboons/macaques. Cortical surfaces of a typical baboon and macaque brain registered in the baboon and MNI standard spaces show that the ventrolateral prefrontal cortex is characterized by the presence of *I-AS* (the homolog of the hominids *IPRS*, in yellow) just in front of *CS* (in green) and the inferior frontal dimple (the homolog of the human *IFS*). Parasagittal sections at the anteroposterior levels represented by dotted lines on the cortical surfaces show that, in cercopithecid monkeys, *CIRC* remains posterior to *I-AS*, showing thus the absence of a PFOp. *CS*, central sulcus; *IPRS*, inferior precentral sulcus; *I-AS*, inferior part of the arcuate sulcus; *CIRC*, circular sulcus; *IFS*, inferior frontal sulcus; *D-FO* and *V-FO*, dorsal and ventral part of the fronto-orbitalis sulcus; *AALF* and *HALF*, ascending and horizontal rami of the lateral fissure.

With the expansion of the frontal cortex in primates, the lateral frontal cortex folds over the insula, which is dorsally limited by the circular sulcus (*CIRC*, Fig. 1a–d, *see the List of abbreviations*). The buried part that is lateral to the insula is the frontal operculum. In the human brain, it extends both in the prefrontal and the premotor/motor cortex (Fig. 1a). We shall refer to its rostral part as the prefrontal extent of the frontal operculum (PFOp). Indeed, PFOp can be observed at the level of the ventrolateral prefrontal cortex, located below the inferior frontal sulcus and in front of the inferior precentral sulcus (*IPRS*). At this level, the ascending anterior ramus of the lateral fissure (*AALF*, the rostral border of the classical Broca’s area, i.e., cytoarchitectonic area 44) is intricately folded to the point that it joins *CIRC*, creating a complete opercularization and thus an additional buried gyrus between Broca’s area and the anterior insula enlarges the PFOp, making it more complex (Fig. 1a). The cytoarchitectonic organization of the PFOp in the human brain indicates that it can be subdivided into four areas (Op7, Op8, Op9, Op10) and is thought to belong to the larger Broca’s complex, which also includes the classic ventrolateral prefrontal cortical areas Brodmann areas 44 and 45 (Fig. 1a)^8^. The emergence of PFOp is of critical importance because the horizontal ascending ramus of the lateral fissure (*HALF*) is located in the most rostral Op region (Op10), thereby creating the fold that defines the pars triangularis. From the lateral surface of the frontal cortex, the presence of *HALF* can therefore be seen as a marker of the presence of PFOp (Fig. 1a). By contrast, the ventrolateral prefrontal cortex of chimpanzees and other great apes is characterized only by the presence of the dorsal part of the sulcus fronto-orbitalis (*D-FO*) (Fig. 1b)^9–14^. Note that the sulcus fronto-orbitalis is composed of two parts: 1) the ventral part (*V-FO*) running on the orbital surface, and 2) the dorsal part running on the ventrolateral prefrontal surface (*D-FO*). *D-FO* appears to be the homolog of the human *AALF* as both sulci limit the cytoarchitectonic Brodmann’s area 44 from area 45^15,16^. A recent study has additionally shown that *D-FO* can be a single sulcus or bifurcated (Fig. 1b), and that chimpanzees displaying a bifurcated *D-FO* have better orofacial motor control abilities, as measured by the ability to produce Attention-Getting (AG) sounds^17,18^, compared with chimpanzees that did not produce such sounds^14^. AG sounds are produced by captive and wild chimpanzees to attract the attention in an otherwise inattentive social audience and are of interest because they are used intentionally and referentially, and they require that the apes develop voluntary control of their oro-facial musculature and larynx. They thus reflect the emergence of critical cognitive and motor abilities for the evolution of speech^17,19–22^. Finally, cercopithecid monkeys display no sulcus below the homolog of the human inferior frontal sulcus, i.e., the inferior frontal dimple^7^ (Fig. 1c, d), suggesting that they do not present a PFOp.

Recent paleoanthropological studies suggest that the *HALF* sulcus can be observed only on endocasts of later hominins from the genus *Homo*, but not earlier australopiths and chimpanzees (*Pan troglodytes*)^23^. Furthermore, it has been hypothesized that some aspects of modern symbolic behavior and language capacities appeared relatively late in the archeological record, being evident only in Neanderthals and archaic *Homo sapiens*^24^. This suggests a critical role of PFOp in the emergence of speech, and the absence of this region in other nonhuman primate species and hominin ancestors with smaller brain sizes. The present study thus aimed to confirm that cercopithecid monkeys (macaques and baboons) do not have a PFOp, identify whether great apes (chimpanzees) display a PFOp precursor, and whether orofacial motor abilities in chimpanzees relate to sulcal organization within this region. We show that only humans present a PFOp with a complete opercularization (i.e., *AALF* joining *CIRC*), and that chimpanzees displaying a sulcal configuration the closest to human-like opercularization have also increased ability to produce AG sounds. By contrast, cercopithecid monkeys do not present any form of PFOp. We thus argue for the importance of PFOp in the evolution of speech abilities.

## Results

### Presence of PFOp in humans, chimpanzees, and cercopithecid monkeys

**Fig. 2:**
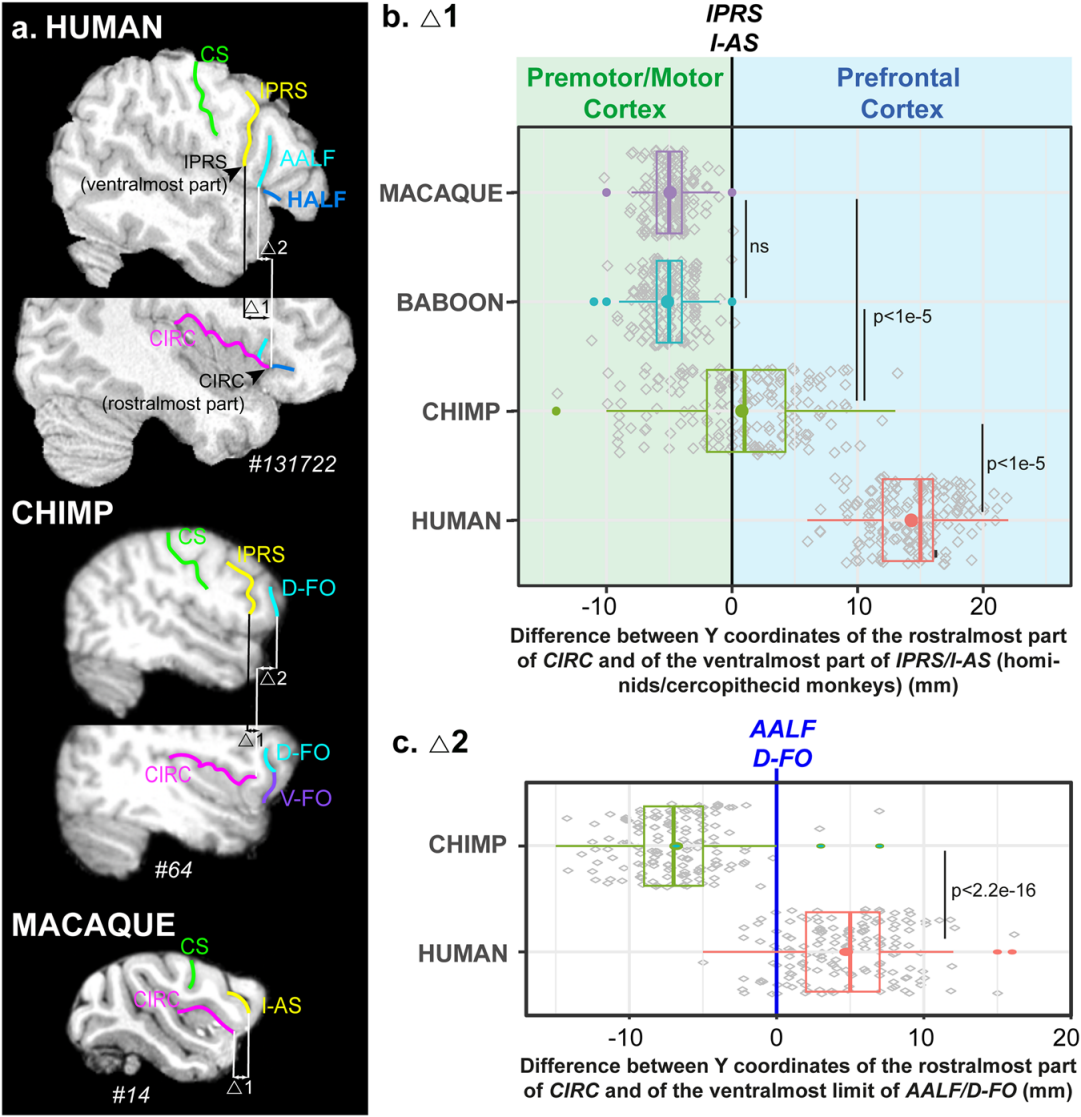
Quantitative assessment of the location of the rostralmost part of *CIRC* in humans, chimpanzees, baboons and macaques. **a** Typical examples from each species showing the quantitative measures on parasagittal sections at different medio-lateral levels. Two measures were performed in each hemisphere of each primate species: **b** the difference between the anteroposterior Y coordinates of the rostralmost part of *CIRC* and the ventralmost part of *IPRS* in humans and chimpanzees and *I-AS* in cercopithecid monkeys; **c** only in humans and chimpanzees, the difference between the anteroposterior Y coordinates of the rostralmost part of *CIRC* and the ventralmost part of *AALF* in human and *D-FO* in chimpanzees (represented by a blue line). Results are shown on boxplots, bold lines and dots represent the median and the mean of the distribution, respectively. Colored circles represent outliers. Gray diamonds represent individual data points. Statistical significant GLMM and Tuckey post-hoc results are shown. Results indicate that only humans display a complete PFOp reaching *AALF*, chimpanzees displaying a precursor of PFOp that does not reach *D-FO*. PFOp is absent in cercopithecid monkeys. *CS*, central sulcus; *IPRS*, inferior precentral sulcus; *I-AS*, inferior part of the arcuate sulcus, *CIRC*, circular sulcus, *D-FO* and *V-FO*, dorsal and ventral part of the fronto-orbitalis sulcus; *AALF* and *HALF*, anterior ascending and anterior horizontal rami of the lateral fissure.

We assessed whether *CIRC* extended to the prefrontal cortex, which would be indicative of the presence of PFOp, on anatomical T1 MRI scans of 80 brains of macaques, baboons, chimpanzees, and humans. We first manually labeled the lateral frontal sulci (i.e., in all primates: CS and *LF; in human: IPRS, AALF, HALF, IFS; in chimpanzee: IPRS, D-FO, IFS*; in cercopithecid monkeys: *I-AS*, inferior frontal dimple) and the sulcus limiting dorsally the insula (i.e., *CIRC*). We then identified in each hemisphere of each species the difference between the Y coordinate of the rostral limit of the circular sulcus and the Y coordinate of the ventral-most level of the inferior part of *IPRS/I-AS* in the brains (Fig. 2a)^7^. Results revealed main differences between species (p < 2.2e-16, F = 997.7, NumDF = 3, DenDF = 325.3, Generalalized Linear Mixed-effects Model -GLMM-): whereas only humans display the rostral-most part of *CIRC* systematically located anterior to *IPRS* (median = 15 mm anterior to *IPRS*), it reaches the level of *IPRS* in chimpanzees (median = 1 mm anterior to *IPRS*), and it never reaches the level of *I-AS* (i.e., the homolog of hominids *IPRS*^7^) in baboon and macaque brains (median = 5 mm posterior to *I-AS*) (Fig. 2b). Note that this pattern was similar in left and right hemispheres in all nonhuman primates (Chimpanzee: F = 0.5, NumDF = 1, DenDF = 79, Baboon: F = 3.33, NumDF = 1, DenDF = 79, Macaque: F = 0.6, NumDF = 1, DenDF = 79, ns at p < 0.05, GLMM). In human brains, CIRC was slightly more anterior in the left than in the right hemisphere, suggesting an enlargement of PFOp in the left hemisphere (p < 0.009, F = 7.1, NumDF = 1, DenDF = 79, GLMM). Furthermore, whereas the rostral-most limit of *CIRC* largely reaches the level of *AALF* in humans (i.e., median = 5 mm anterior to *AALF*), it never reaches the antero-posterior level of *D-FO* in chimpanzees (i.e., median = 7 mm posterior to *D-FO*) (human versus chimpanzee: p < 4.4e-6, F = 22.6, NumDF = 1, DenDF = 157.9, GLMM) (Fig. 2a, b). These results demonstrate that only humans have a fully developed PFOp, with a complete opercularization between *AALF* and *CIRC*, i.e., the critical anatomical element for the formation of a full Broca’s complex composed of a pars opercularis and triangularis (Fig. 1). Chimpanzees display a precursor of PFOp with *CIRC* that is beginning to invade the prefrontal cortex, but it never reaches *D-FO* (i.e., the homolog of the human *AALF*), preventing a complete opercularization and the formation of a full Broca’s complex. Finally, the PFOp is totally absent in cercopithecid monkeys. Note that, in humans, the complete opercularization in PFOp occurs at the merging point between *AALF* (the homolog of the monkey *D-FO*) and *CIRC*, and that *CIRC* thus never joins the orbitofrontal cortical surface where *V-FO* lies in chimpanzees. As such, it is reasonable to hypothesize that the configuration in which *V-FO* does not join *CIRC* is the configuration that is closest to the one observed in the human brain. Note that Figs. S1–S4, respectively display additional examples of the sulcal configuration of this region in typical human (n = 3 brains/6 hemispheres), chimpanzee (n = 10 brains/20 hemispheres), baboon (n = 8 brains/16 hemispheres), and macaque (n = 8 brains/16 hemispheres) brains.

### Characterization of PFOp local morphology and its impact on orofacial motor abilities in chimpanzees

**Fig. 3:**
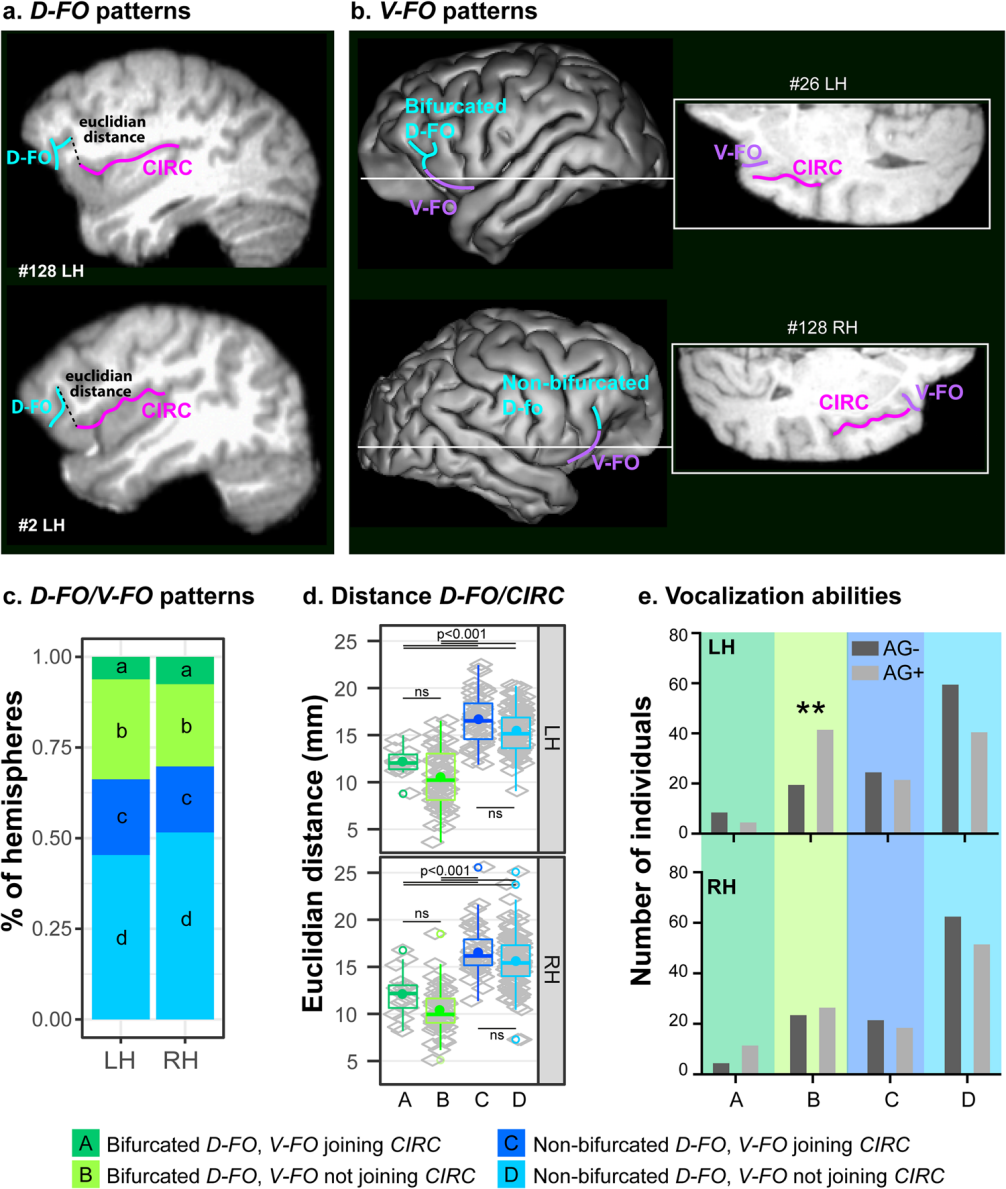
Patterns *D-FO/V-FO* in chimpanzees and relationships with vocalizations. **a** *D-FO*: typical examples of chimpanzees displaying a bifurcated and a non-bifurcated *D-FO* are presented on sagittal sections. **b** *V-FO*: typical examples of chimpanzees displaying a *V-FO* joining or not joining the *CIRC* are presented on cortical surfaces and horizontal sections. **c** Percent of hemispheres displaying each pattern in left (LH) and right (RH) hemispheres: four patterns are observed bilaterally: a bifurcated *D-FO* and a *V-FO* joining *CIRC* (dark green), a bifurcated *D-FO* and a *V-FO* not joining *CIRC* (light green), a non-bifurcated *D-FO* and *V-FO* joining *CIRC* (dark blue), and a non-bifurcated *D-FO* and *V-FO* not joining *CIRC* (light blue). **d** Euclidean distance between the most caudal part of *D-FO* and the most rostral part of *CIRC* (as displayed in (**a**)): The Euclidean distance is the shortest in hemispheres displaying a bifurcated *D-FO*, regardless of *V-FO* joining or not the *CIRC*. Results are shown on boxplots, bold lines and dots represent the median and the mean of the distribution, respectively. Colored circles represent outliers. Gray diamonds represent individual data points. **e** Vocalization abilities (AG + /AG−) in chimpanzees displaying the four patterns described in (**c**) and (**d**): chimpanzees displaying in the left hemisphere a bicurcated *D-FO* and a *V-FO* not joining *CIRC* are mostly AG + (at p < 0.005). LH/RH: left/right hemisphere; ns, non-significant at p < 0.05.

To characterize PFOp in chimpanzees, we used a large neuroimaging dataset from the National Chimpanzee Brain Resource (n = 225/450 brains/hemispheres, http://www.chimpanzeebrain.org/). The assessment of the morphology of this region in this dataset revealed that, although *CIRC* never joins *D-FO* (Figs. 2c and 3a), it joins *V-FO* in 26.4% of hemispheres (Fig. 3b). Thus, four sulcal patterns were observed in both hemispheres (Fig. 3c, d): 1. A bifurcated *D-FO* and a *V-FO* joining *CIRC* (6.9% of hemispheres), 2. A bifurcated *D-FO* and *V-FO* not joining *CIRC* (25.1% of hemispheres), 3. A non-bifurcated *D-FO* and *V-FO* joining *CIRC* (19.6% of hemispheres), 4. A non-bifurcated *D-FO* and *V-FO* not joining *CIRC* (48.4% of hemispheres). No difference was observed in the occurrence of these patterns in the left versus the right hemisphere (F = 0.9889, NumDF = 1, DenDF = 224.12, p < 0.3211, Generalized Linear Mixed-effects Model -GLMM- with hemispheres as fixed effect and animal ID as random effect, formula: pattern ~ hemisphere + (1 | ID)).

We next assessed whether individual differences in the ventrolateral prefrontal sulcal morphology were associated with aspects of chimpanzee vocal control during communication that may have served as pre-adaptations to the emergence of speech. We calculated the Euclidean distance in individual brains and hemispheres between the dorsal caudal-most part of *D-FO* and the rostral-most part of *CIRC* (Fig. 3a). A GLMM with patterns and hemispheres as fixed effects and animal ID as random effect (i.e., Euclidean distance ~ pattern * hemisphere + (1 | ID)) demonstrated that the Euclidean distance is the shortest in hemispheres displaying a bifurcated *D-FO*, whether *V-FO* joins or does not join *CIRC* (p < 2e-16, F = 128.8, NumDF = 3, DenDF = 434.2, GLMM, Fig. 3d). Note that the Euclidean distance was similar in left and right hemispheres (ns at p > 0.05, F = 0.04, NumDF = 1, DenDF = 272.5, GLMM), and that no interaction was observed between the pattern and hemisphere (ns at p > 0.05, F = 0.16, NumDF = 3, DenDF = 364.2, GLMM). By shortening the distance between *D-FO* and *CIRC*, the presence of a bifurcated *D-FO* is associated with a morphological pattern that appears to resemble more closely the opercularization of this ventrolateral prefrontal region in the human brain.

We assessed whether the Euclidean distance was related to orofacial motor abilities, regardless of the hemisphere and of the pattern observed in the ventrolateral prefrontal cortex. Results revealed a trend of the Euclidean distance on AG sound production with increased orofacial motor abilities associated with decreased Euclidean distance (F(1, 218) = 3.455, p = .064, GLM with AG + /- as factors and distance as fixed effect).

Finally, we hypothesized that, if an important anatomical contribution to speech emergence is the opercularization of *D-FO* with *CIRC*, chimpanzees displaying the pattern “Bifurcated *D-FO* and *V-FO* not joining *CIRC*” should be more likely to produce AG sounds, whereas all other patterns, including patterns in which *V-FO* joins *CIRC*” should not impact this ability. The results indeed revealed that it is the only pattern associated with increased use of AG sounds (Fig. 3e). An unexpected and important result is that this is the case only if this pattern is observed in the left hemisphere (*X*^2^ = 12.65, p < 0.005) but not the right hemisphere (*X*^2^ = 4.26, p = 0.235, ns). Indeed, 70% of chimpanzees displaying this pattern in the left hemisphere reliably produced AG sounds, suggesting an emergence of a lateralization in the control of oro-facial musculature and vocalizations. These results add further support to the finding from Hopkins et al.^14^ showing that the presence of a bifucarted *D-FO* in the left or in both hemispheres was associated with better orofacial motor control.

### Impact of the coincidence of the presence of a PCGS

**Fig. 4:**
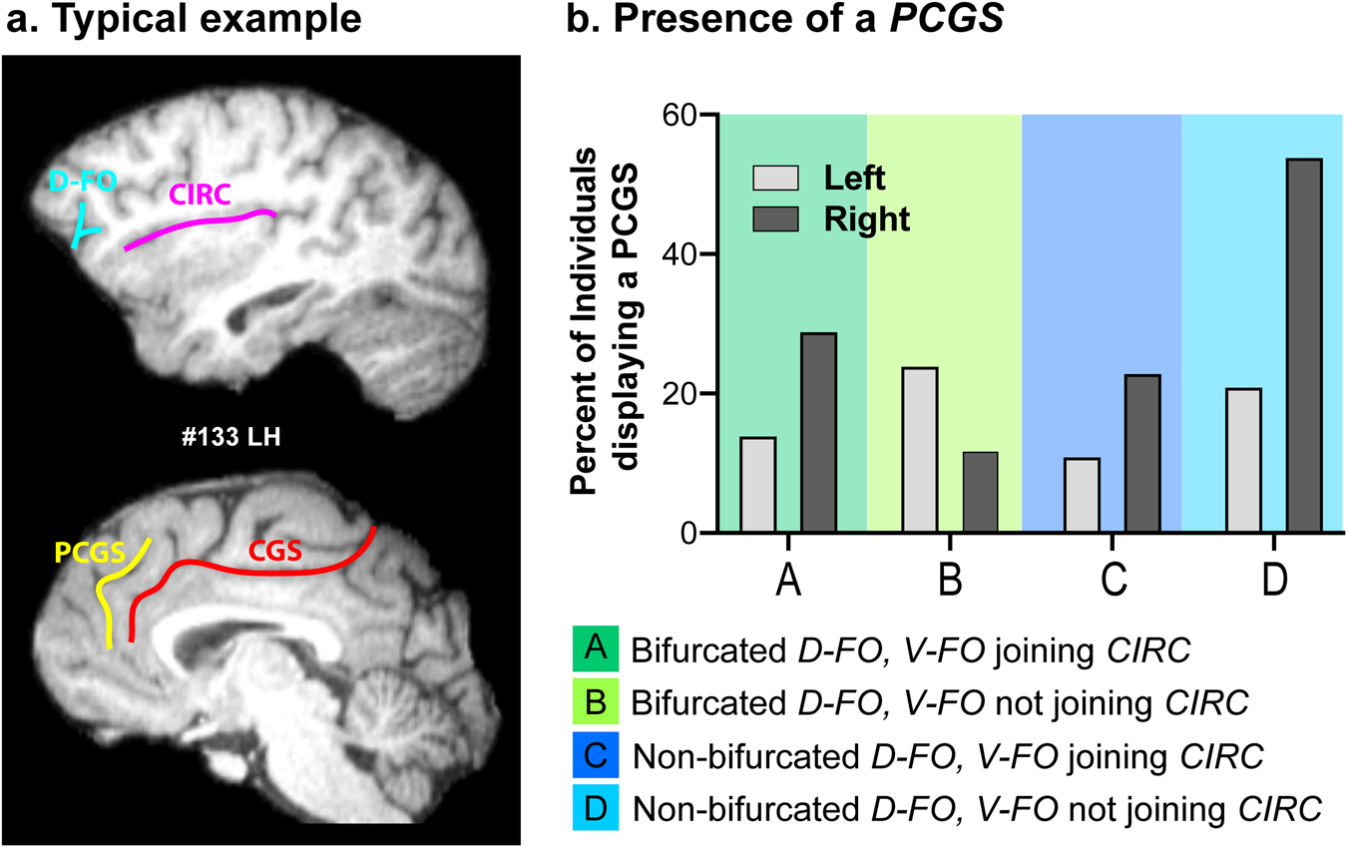
Combined presence of a *PCGS* with the various patterns observed in the ventrolateral prefrontal cortex. **a** Typical example of a chimpanzee displaying both a bifurcated *D-FO* and a *PCGS*. **b** Percent of chimpanzees displaying a *PCGS* in each one of the four configurations observed in the ventrolateral prefrontal cortex in the left and right hemispheres. Data show that the frequency of occurence of a *PCGS* in the left hemisphere is higher than in the right hemisphere only when the *D-FO* is bifurcated and the *V-FO* does not join *CIRC* in the ventrolateral prefrontal cortex.

We have recently shown in the same chimpanzee dataset that the presence of a paracingulate sulcus (*PCGS*) in the medial frontal cortex in the left hemisphere was associated with greater ability to produce AG sounds^25^. We therefore assessed whether chimpanzees displaying a bifurcated *D-FO* were also more likely to have a *PCGS* in both hemispheres. Results showed that the only pattern associated with an increased presence of a *PCGS* in the left compared to the right hemisphere was the pattern “Bifurcated *D-FO* with *V-FO* not joining *CIRC*” (Fig. 4a/b). All the other patterns were associated with an increased frequency of occurrence of a *PCGS* in the right than in the left hemisphere. The assessment of the ability to produce AG sounds in the individuals displaying this specific configuration in the left hemisphere (i.e., 15 chimpanzees) revealed that 60% (9/15) of them were AG+. Note that only 2 individuals displayed this configuration in the right hemisphere and only one of these was AG+. Finally one individual displayed bilaterally both a *PCGS* and bifurcated *D-FO* with a *V-FO* not joining *CIRC* and he was AG+. Although these results should be taken cautiously given the small sample presenting both the presence of a *PCGS* and a bifurcated *D-FO*, results suggest that the combination of the presence of these two sulcal patterns might be related to improved orofacial motor abilities.

## Discussion

The present study demonstrates that the human brain displays a unique feature in the ventrolateral prefrontal cortex compared with chimpanzees, i.e., a fully opercularized PFOp region, located adjacent and ventromedial to the classical Broca’s area. In contrast to cercopithecid monkeys in which the PFOp is absent, chimpanzee brains present precursors of the sulcal configuration at the origin of the formation of a human-like PFOp: 1) a circular sulcus reaching the caudalmost part of the prefrontal cortex, and 2) a *D-FO* sulcus, i.e., the homolog of the human *AALF*^15^. Importantly, chimpanzees that display the most human-like PFOp, particularly in the left hemisphere, have greater oro-facial and vocal motor control abilities. Relatedly, recent paleoanthropological studies have shown that human-like PFOp morphology (i.e., presence of the *HALF* sulcus) can be also observed on endocasts of certain smaller-brained hominins of the genus *Homo* (*Homo naledi, Homo erectus*) but not in earlier hominins from the genus *Australopithecus* (*Autralopithecus sediba, Australopithecus africanus*)^23^, suggesting that human-like PFOp morphology originated at some point after the first emergence of the genus *Homo* over 2 million years ago, and is not simply linked to brain size expansion in later species such as *Homo sapiens* and Neanderthals, around 400 000 to 800 000 years ago^26^. Combined with our results, these findings support the view that PFOp is a recently evolved feature of human cortical structure (perhaps limited to the genus *Homo*) associated with cognitive and motor functions evident in modern speech abilities.

**Fig. 5:**
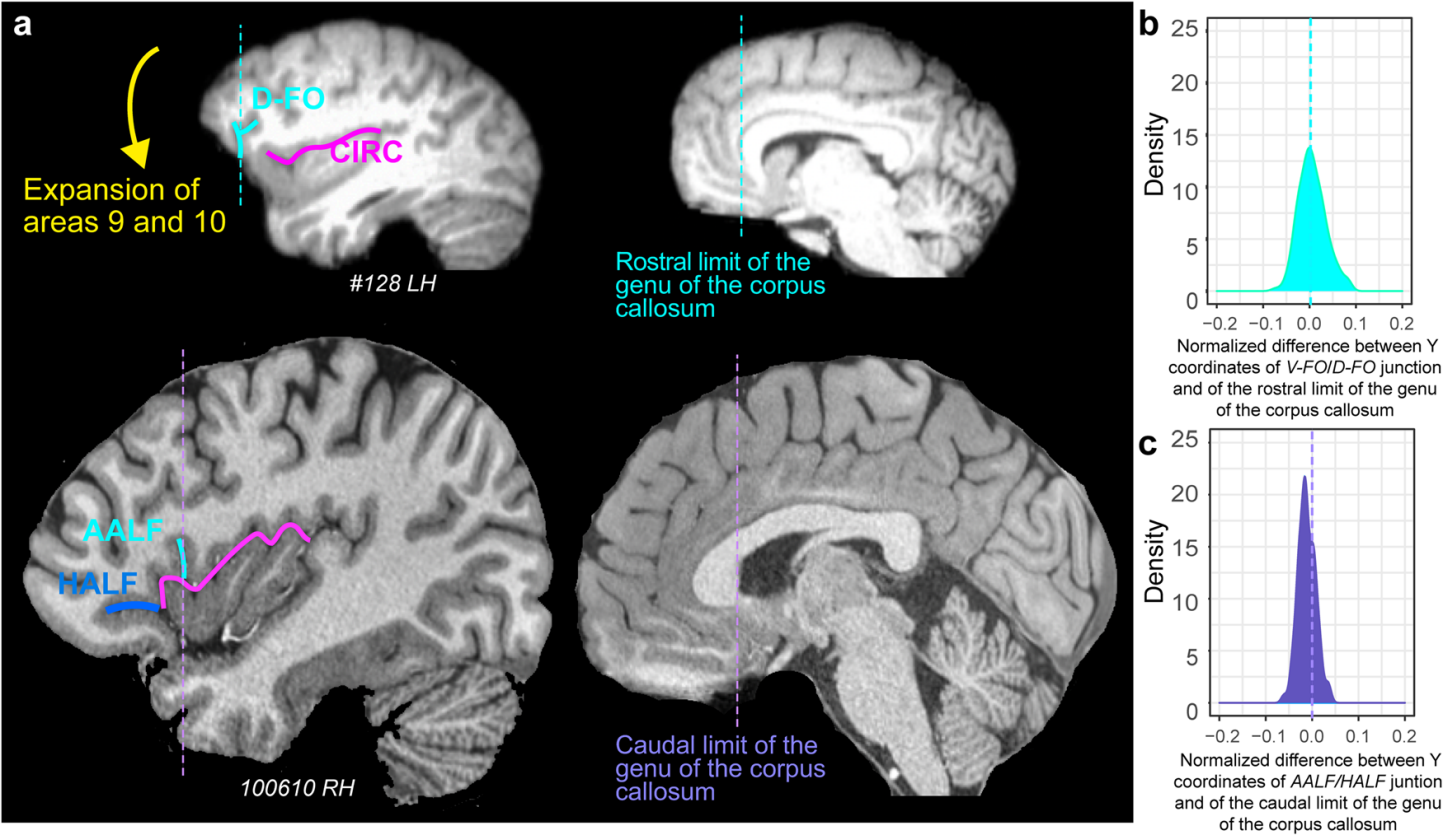
Evolution of the ventrolateral prefrontal cortical region. **a** As shown on a sagittal view of a typical chimpanzee hemisphere, the expansion of areas 9 and 10 (indicated by the yellow arrow) would allow a downward shift of *D-FO* and consequently render possible the creation of a frontal operculum as observed in the human brain (left side brains). Right side brains display the location of the rostral limit of the genu of the corpus callosum in chimpanzee and the caudal limit of the genu of the corpus callosum in human brains. **b**, **c** Density of the difference between the Y coordinates of **b**) in chimpanzee the *D-FO/V-FO* junction and of the Y coordinate of the rostral limit of the genu of the corpus callosum (top representation), **c** in human the *AALF/HALF* junction and of the Y coordinate of the caudal limit of the genu of the corpus callosum (lower representation). These differences were normalized in relation to the brain size (total antero-posterior extent). The 0 value corresponds to the Y level of the rostral (top representation) and caudal (lower representation) limit of the genu of the corpus callosum in standard brains. Results show that whereas the chimpanzee specific *D-FO/V-FO* junction is located at the level of the rostral limit of the genu of the corpus callosum, the human-specific *AALF/HALF* junction is located caudally, i.e., at the level of the caudal limit of the genu of the corpus callosum.

We have recently shown that the sulcal organization of much of the frontal cortex (i.e., including the posterior frontal cortex, the dorsolateral and medial prefrontal cortex, and the frontopolar cortex) is highly conserved in chimpanzees and humans^7^. Notably, the ventrolateral prefrontal cortex is the region that differs most significantly in sulcal folding between humans and chimpanzees. Together with the present study, these findings demonstrate that the ventrolateral prefrontal cortical region has undergone substantial modification in human brain evolution. Comparative studies in primates have suggested differential expansion of the medial and lateral prefrontal cortex in humans^3,6,27,28^. In line with these results, the present study suggests that the only way to render physically possible the joining of the dorsal caudalmost part of *D-FO* and the rostralmost part of *CIRC* is a prior expansion of these areas (Fig. 5a). This is also supported by the analysis of the location of the ventralmost part of *D-FO* in chimpanzee and the ventralmost part of *AALF* in the human brain. Indeed, our results show that, in chimpanzees, *D-FO* is spatially aligned to the rostral limit of the genu of the corpus callosum (Fig. 5b) whereas, in humans, *AALF* is aligned to a more caudal anatomical landmark, i.e., the caudal limit of the genu of the corpus callosum (Fig. 5c). Collectively, these results strongly suggest that the expansion of prefrontal cortical areas 9 and 10 is associated with the downward and backward shift of *D-FO*, creating the conditions for the emergence of PFOp, the pars triangularis, and a fully developed Broca’s area complex.

According to Amunts et al.^8^, in humans, PFOp is occupied by cytoarchitectonic areas Op7, Op8, Op9 and Op10, whereas, caudal to IPRS, the frontal ‘premotor/motor’ operculum is occupied by areas Op6 and Op4. Our sulcal analysis across primates revealed that PFOp is absent in macaques and baboons, whereas some precursors emerge in chimpanzees. Importantly, macaques display a frontal operculum^29^, called ProM^30^, or subdivided in PrCO and GrFO^31^, located posterior to the inferior arcuate sulcus, in line with our observations. This region appears to be connected to mouth-related ventral premotor cortex and somato-sensory areas^32^ and plays a role in sensorimotor control of jaw, oropharyngeal, and laryngeal movements^33,34^. Given the common location of PrCO in macaque and Op6 and Op4 in human, i.e., posterior to the inferior part of the arcuate sulcus in macaque and the inferior precentral sulcus in human brains, we hypothesize that the macaque frontal operculum corresponds to the caudal frontal operculum areas Op7 and Op8 in humans^8^. Future comparative cytoarchitectonic and connectivity analyses should be performed to validate this hypothesis, and should be extended to chimpanzee brains.

The present study, and others, therefore demonstrate the sulcal characteristics that have evolved in the human frontal cortex since the split from their last common ancestor with chimpanzees^6^. Whereas other uniquely human landmarks, such as the rostro-perpendicular paracingulate sulcus, reflect a local expansion of prefrontal areas 9 and 10, the present results show a profound reorganization of the ventrolateral prefrontal cortex. In addition, our study points toward a leftward asymmetry of the homolog of the frontal network involved in speech abilities by showing that chimpanzees displaying a bifurcated *D-FO* with a *V-FO* not joining *CIRC* in the left hemisphere, also possess a *PCGS*, and have increased abilities to produce AG sounds. Interestingly, the presence of either a bifurcated *D-FO* with a *V-FO* not joining *CIRC* (present results) or a *PCGS*^25^ specifically in the left hemisphere are both linked with improved orofacial motor abilities. The leftward asymmetry in this network is dominant in the human brain, with an enlarged Broca’s complex and a higher frequency of occurrence of a *PCGS* in the left compared to the right hemisphere^35,36^. Importantly, this network is thought to be critically involved in the cognitive control of vocalizations and to have considerably evolved since the split between the cercopithecid monkeys and human lineages^37^. One can thus hypothesize that the combination of these particular sulcal characteristics in the cingulate and the ventrolateral prefrontal cortex allows a particular connectivity profile that provides an advantage for speech abilities. Future studies may test this hypothesis by means of the analysis of diffusion tensor imaging data in chimpanzee brains for which their orofacial motor control abilities are known. Our results add to previous studies assessing morphological differences often associated with the emergence of modern speech abilities showing a leftward asymmetry of the ventrolateral frontal cortex^38^ and the *planum temporale*^39,40^, and suggest that some aspects of the leftward lateralization of this network dominating the human brain anatomo-functional organization emerged as early as the split between great apes and human lineages 6 million years ago, or even before^40^.

Concomitantly, the paleoanthropological analyses of the endocranial morphology of fossils also suggest that the complete opercularization of the PFOp only occurs in the genus *Homo*^23^, roughly synchronized in time with increasing evidence for the beginnings of modern speech capacities^24^. Paleoanthropological studies have shown that Neanderthals might have possessed the ability to produce symbolic behaviors that support advanced language abilities^41–44^. Additionally, the structures of the middle and outer ears are very similar in Neanderthal and modern Homo sapiens. They support the same sound power transmission (i.e., as measured by the Occupied BandWidth -OBW-), a proxy for auditory sensitivity, through the outer and middle ears to the inner ear^45,46^. Specifically, the observed OBW is known to allow the distinction of high-frequency consonants in modern Homo Sapiens, a critical aspect of human speech for the identification of word meaning after further decoding by dedicated neuronal networks^24,47^.

These results push towards a shift in paradigm in assessing the normal and abnormal functioning of the speech network in humans^48^. Indeed, the network in the human brain is composed of regions that have clear homologs in non-speaking primates, such as Broca’s area^49^. Yet, these studies have not addressed the question of the uniqueness of the human PFOp. Given the tight correlation between the evolutionary time of the appearance of PFOp as suggested by endocranial morphology, and of an auditory system allowing the brain to distinguish acoustic features that facilitate speaker identity vs the processing of word meaning, two hypotheses emerge: 1) PFOp optimizes the anatomo-functional relationships between Broca’s area (responsible for speech production) and auditory superior temporal cortical areas (responsible for the analysis of auditory information); 2) PFOp supports complex manipulation of cognitive representations required for speech (e.g., top-down regulation of selective retrieval of information from parietal and temporal cortical areas).

The first hypothesis finds support in the architecture of the white matter tracts that connect the ventrolateral frontal cortex and the superior temporal cortex. Specifically, tract-tracing studies in macaques have shown that two separate monosynaptic and bi-directional auditory pathways between the temporal lobe to the ventrolateral frontal cortex exist: 1) the dorsal pathway, which refers to the arcuate fasciculus (AF) connecting area 44 with the dorsal posterior temporal region (Wernicke’s area), and 2) the ventral pathway, which refers to the temporo-frontal extreme capsule fasciculus (TFexcF) connecting area 45 with the anterior parts of the superior and middle temporal gyri (i.e., the parts anterior to the sulcus acousticus, and which thus do not belong to Wernicke’s area); the latter connection is via the anteriormost part of the extreme capsule, i.e., the white matter between the anterior insula and the claustrum^16,50–56^. Note that area 44, in addition of receiving auditory information from the AF, also receives high-level orofacial information from the supramarginal gyrus via the Superior Longitudinal Fasciculus III (SLF III), and the overall integration of information from these regions is critical to generate proper speech output^57–61^. These two pathways were also observed in human brains, suggesting the emergence of basic processing in nonhuman primates that expands in the human brain, leading to speech^16,37,49,53,62–68^. The argument of the dual origin of language is built on these data and posits that the dorsal stream is involved in phonological sublexical processing of linguistic information (i.e., mapping of sound-to-articulation), and the ventral stream is involved in the semantic processing of linguistic information (i.e., mapping sound-to-meaning)^60,69,70^. However, whereas to the best of our knowledge, TFexcF appears to have a similar organization in human and macaque brains^50,51,60,71^, AF presents some qualitative and quantitative differences across primate species^4,62,71,72^. First, in human brains, the AF runs from the ventrolateral prefrontal cortex to the temporo-parietal junction and then turns around the posterior end of the lateral fissure to reach the posterior part of the superior temporal gyrus and adjacent sulcus, i.e., the classical speech comprehension region (Wernicke’s region), as well as the middle and inferior temporal gyrus^62,63^. In macaque monkeys, area 44 connects with the cortex within the adjacent superior temporal sulcus, i.e., in the sulcal part of area Tpt which occupies the posterior part of the superior temporal gyrus and sulcus but does not reach the middle and inferior temporal gyrus^55,62,63^. Second, the AF displays a strong leftward asymmetry in the human brain, but it appears not to be the case in macaques^62,63^. Note, however, that the absence of asymmetry of the AF in non-human primates would need to be confirmed in larger datasets. By contrast, the ventral pathway does not display a leftward asymmetry in both human and chimpanzee brains^71^. Third, AF fibers are weaker in macaque than in chimpanzee than in human brains, suggesting weak anatomical connections between Broca’s area and auditory temporal area through this fasciculus in non-human primates^4,62,64,72^. Finally, it has been shown that the configuration of the AF in humans is setup during development: whereas the AF present a similar extent in newborn human infants and monkeys, it develops during childhood and this development co-occurs with the onset of language acquisition^73^. These data, together with our results showing that chimpanzees displaying morphological features closest to the opercularization in the left hemisphere have greater vocal control abilities, converge towards the importance of the emergence of PFOp. Given the medial strategic location of PFOp in human brains, extending from the caudal level of area 44 (*IPRS*) to the level of the junction between areas 44 and 45^8^, it may optimize the connectivity between Broca’s region and temporal cortical areas, and allow the integration of information coming from the ventral TFexcF and the dorsal AF paths. Both cases, associated with the emergence of PFOp in the human brain, may provide an evolutionary advantage for the emergence of speech and language abilities.

The second hypothesis finds support from neuroimaging studies pointing towards a specific involvement of PFOp in the initiation of speech/language^74–76^ and in syntactic constructions^77–84^, i.e., the ability to manipulate abstract relationships between words and combine them together to form sentences^85^. Note that these data reinforce the hypothesis that PFOp belongs to the dorsal pathway, as this pathway is known to be associated with syntactic processing^78,86,87^. Other support for this hypothesis comes from lesion studies showing that patients with large lesions in Broca’s area including PFOp in the dominant hemisphere display aphasia and apraxia^88–90^. Note that, to the best of our knowledge, the only study identifying a specific lesion in the frontal operculum, a region located in the precentral gyrus, just posterior to the PFOp, is Mitani et al.^91^ who have shown that prominent iron accumulation in this region in a patient with amyotrophic lateral sclerosis was specifically associated with speech apraxia, as also suggested by Tomaiuolo et al.^92^. Thus, by its additional proximity with frontal cognitive and premotor areas, the PFOp may act as a functional hub to integrate sensory, motor and cognitive information required for complex speech production^93,94^. Future studies perturbing specifically the PFOp in humans, by mean of transcranial ultrasound stimulation (TUS) technology, and assessing its specific impact on the whole-brain functional connectivity and speech-related functions, would identify the causal role of PFOp.

Finally, certain other anatomical differences between human and other primate brains are thought to reflect specializations important for speech evolution. For instance, enhanced connectivity in the laryngeal motor network in humans compared to other primates has been suggested to reflect increased laryngeal control essential for speech evolution^95,96^. Shifts in the functional coupling between medial and lateral frontal cortex with auditory cortex have also been hypothesized to reflect an improved ability to manipulate auditory information in humans compared with other primate species^3^. One may hypothesize that the presence of a PFOp optimizes these functions. To test this hypothesis, future neuroimaging studies may identify the anatomical and functional connectivity of PFOp in humans, as well as its role in these functions critical for speech.

In summary, our study shows the progressive setup of PFOp through evolution (i.e., from its absence in cercopitecid monkeys, to its incomplete opercularization in chimpanzees, and finally to its full opercularization in humans), and its relation with improved orofacial/vocal control abilities, thus providing a possible explanation of changes that occurred to enable modern speech abilities.

## Methods

Neuroimaging T1 anatomical data of 80 human, 225 chimpanzee, 80 baboon, and 80 macaque brains were analyzed.

### Human subjects

High-resolution anatomical scans of human brains were obtained from the Human Connectome Project database (humanconnectome.org). Only data from subjects displaying no family relationships were analyzed. The participants in the HCP study were recruited from the Missouri Family and Twin Registry that includes individuals born in Missouri^97^. Acquisition parameters of T1 anatomical scans are the following: whole head, 0.7mm^3^ isotropic resolution, TR = 2.4 s, TE = 2.14 ms, flip angle = 8° (more details can be found on https://humanconnectome.org). The full set of inclusion and exclusion criteria is detailed elsewhere^97^. Briefly, the HCP subjects are healthy individuals free from major psychiatric or neurological illnesses. They are drawn from ongoing longitudinal studies^97–99^, where they had received extensive assessments, including the history of drug use, emotional, and behavioral problems. The experiments were performed in accordance with relevant guidelines and regulations and all experimental protocols were approved by the Institutional Review Board (IRB) (IRB # 201204036; Title: ‘Mapping the Human Connectome: Structure, Function, and Heritability’). All subjects provided written informed consent on forms approved by the Institutional Review Board of Washington University in St Louis.

### Chimpanzees

High-resolution T1-weighted magnetic resonance image scans of chimpanzee brains were obtained from the National Chimpanzee Brain Resource (NCBR) (www.chimpanzeebrain.org). Note that no new data were collected specifically for the purpose of the present study. Chimpanzee data collection was approved by the Institutional Animal Care and Use Committees at Yerkes National Primate Research Center and the National Center for Chimpanzee Care (NCCC) which is part of the University of Texas MD Anderson Cancer Center, and followed the guidelines of the Institute of Medicine on the use of chimpanzees in research.

### Cercopithecid monkeys

High-resolution anatomical scans of baboon brains were obtained from Dr. Adrien Meguerditchian’s laboratory, and macaque brains were obtained from the laboratories of Drs. E. Procyk, C. Amiez, J. Sallet, F. Hadj-Bouziane, and S. Ben Hamed. Data collected initially for studies on macaque monkeys and baboons were conducted under local ethical agreements (licenses from the United Kingdom (UK) Home Office; Provence and Lyon ethics committees) and in accordance with The Animals (Scientific Procedures) Act 1986 and with the European Union guidelines (EU Directive 2010/63/EU).

### Neuroimaging data analysis

Human brains were normalized in the human (http://www.bic.mni.mcgill.ca/ServicesAtlases/HomePage) Montreal Neurological Institute (MNI) standard stereotaxic coordinate system. Chimpanzee brains were normalized in the chimpanzee standard brain developed by Hopkins and Avant^100^. Baboon brains were normalized in the baboon standard brain developed by Dr. A. Meguerditchian^101^ (http://www.nitrc.org/projects/haiko89/). Macaque brains were normalized in the macaque^102^ MNI stereotaxic coordinate systems. Note that normalization of all primate brains consisted of linear registrations, which allows within-species comparisons between brains without altering relationships between sulci and gyri^6^. It is, therefore, unlikely that such processing influences commonality and divergence of sulcal organization observed between species. Normalization of primate brains was performed with SPM12 (https://www.fil.ion.ucl.ac.uk/spm/software/spm12/). Cortical surfaces were created with the pack Morphologist of BrainVisa (https://brainvisa.info/web/morphologist.html).

We first identified the prefrontal cortex in all individuals. This part of the cortex corresponds to the sector of the frontal cortex located anterior to the premotor cortex, and displaying on the caudo-rostral axis a dysgranular to granular cytoarchitectonic structure^103,104^. Both in cercopithecid monkeys (macaques and baboons) and in great ape and human brains, the caudal limit of the frontal cortex is the central sulcus^105–107^. In great apes and humans, the caudal limit of the prefrontal cortex is the posterior part of the medial frontal sulcus (*PMFS-P*), dorsally, and the inferior part of the inferior precentral sulcus (*IPRS-I*), ventrally^7,103,108^. In cercopithecid monkeys, this limit is comprised by the dorsal and the ventral parts of the arcuate sulcus (Fig. 1)^103^, which have recently been suggested to be the homologs of the human *PMFS-P* and *IPRS-I*, respectively^7,108^.

We assessed whether the circular sulcus extends to the prefrontal cortex in all species. Towards that goal, we identified in 80/160 brains/hemispheres for each species the rostral limit of the circular sulcus (i.e., the anteroposterior Y coordinate), which corresponds to the rostral end of the anterior insula. We also identified the ventralmost level of the inferior part of the inferior precentral sulcus (*IPRS-I*) in human and chimpanzee brains and of its homolog in cercopithecid monkeys, i.e., the ventral part of the arcuate sulcus, both corresponding to the limit between the frontal and the prefrontal cortex^7^. We then assessed the difference between these two Y coordinates (Y rostralmost limit of the circular sulcus minus Y ventralmost part of *IPRS-I/I-AS*). Finally, in chimpanzee and human brains, we assessed the difference between the antero-posterior Y level of the rostralmost limit of the circular sulcus with the Y level of the ventralmost part of *AALF* (in humans) and *D-FO* (in chimpanzees).

We then identified, in 80 human brains/160 hemispheres and in 225 chimpanzee brains/450 hemispheres, *CIRC*, that limits the insula dorsally, and the sulci located in the lateral frontal cortex, i.e., the inferior precentral sulcus (*IPRS-I*), and the central sulcus (*CS*). We also identified specifically in the human brain the ascending anterior ramus of the lateral fissure (*AALF*), and in chimpanzee brains the dorsal and ventral parts of the fronto-orbitalis sulcus (i.e., *D-FO* and *V-FO*). Towards that goal, each MRI scan was inspected visually to assess the presence or absence of these sulci in either hemisphere of each normalized brain for each species assessed. We then assessed whether, in human brains, the *AALF* joins *CIRC*, and whether in chimpanzee brains, the *D-FO* and/or *V-FO* joins *CIRC*. Because the results revealed that *D-FO* never joined *CIRC*, we calculated the Euclidean distance between the dorsal caudalmost part of *D-FO* and the rostralmost part of *CIRC* in all chimpanzee hemispheres. Note that when *D-FO* was bifurcarted, we used the dorsalmost part of the caudal branch of *D-FO*. Towards that goal, we extracted the X,Y,Z coordinates of these two latter points (see Fig. 4a) and applied the Euclidean distance formula: sqrt[(Xa-Xb)² + (Ya-Yb)² + (Za-Zb)²].

We thus identified chimpanzees displaying a paracingulate sulcus (*PCGS*), and identified in these subjects the pattern observed in the ventrolateral prefrontal cortex.

Finally, on a subsample of 80/160 human and 80/160 chimpanzee brains/hemispheres, we assessed any relationships that may exist between *D-FO* in chimpanzee and *AALF* in human and particular fixed anatomical landmarks across the species were examined, such as the caudal and rostral limits of the genu of the corpus callosum or the anterior commissure. The relationships between the location of a given sulcus and a particular anatomical landmark across species were examined as follows:

(a) In chimpanzee to assess whether *D-FO* was located at the level of the rostral limit of the genu of the corpus callosum, we calculated, in individual brains, the difference between the Y value of the dorsal caudalmost part of *D-FO* and the Y value of the rostral limit of the genu of the corpus callosum.

(b) In human, to assess whether *AALF* was located at the level of the caudal limit of the genu of the corpus callosum, we calculated, in individual brains, the difference between the Y value of the *AALF/HALF* intersection and the Y value of the caudal limit of the genu of the corpus callosum.

These differences were calculated on the normalized T1 data of these species and were then normalized to take into account the different antero-posterior extents of the brains of the two species, the antero-posterior extent of the human and chimpanzee brains being, respectively, 175 and 110 mm. The normalization performed within species was obtained by dividing the Y coordinate of the sulcus of interest, measured on brains registered linearly in the species-specific standard space, by the antero-posterior extent of the standard brain.

In both analyses, we labeled all the sulci in the human brains and the chimpanzee brains with the exception of *D-FO* in chimpanzee that carried out in a previous study^14^.

### Measuring attention-getting sounds

We tested for associations between the presence of the 4 patterns in each hemisphere in chimpanzee and the use of attention-getting vocalizations (AGs) by the chimpanzees. We also identified the proportion of chimpanzees displaying both a *PCGS* and a bifurcated *D-FO* with *V-FO* not joining *CIRC* in the left hemisphere and the proportion of them displaying increased orofacial motor control. The methods and procedures for classification of subjects as reliably (AG + ) or not reliably (AG-) producing AG sounds has been discussed in detail in previous studies^17,18^.

### Statistics and reproducibility

We tested the influence of hemispheres on the rostro-caudal extent of *CIRC* in all species with the General Linear Mixed Model with hemisphere and species as independent variables and ID as random factor, and we thus performed post-hoc Tukey tests. We tested the influence of hemispheres on the presence of the various patterns observed in the ventrolateral prefrontal cortex of chimpanzees with the General Linear Mixed Model with hemisphere as independent variable and ID as random factor, and then we performed post-hoc Tukey tests. We also tested the influence of these patterns and hemispheres on the Euclidean distance between *D-FO* and *CIRC* with a General Linear Mixed Model with the Euclidean distance as independent variable, patterns and hemispheres as fixed effect, and chimpanzee ID as random factor. Post-hoc Tukey tests were then applied. We also tested the influence of the various patterns and hemispheres on the vocalization abilities with the AG score using Chi-square tests of independence (see source data in supplemental materials). All statistics were performed with R software, R Development Core Team^109^ under R-Studio^110^.

### Reporting summary

Further information on research design is available in the Nature Portfolio Reporting Summary linked to this article.

## Supplementary information

Supplementary Information

Description of Additional Supplementary Files

Supplemental Data 1

Reporting Summary

## Data Availability

Supplemental Data 1 includes the source data underlying Figs. 2–5.
