# Supplementary material for: The relevance of the unique anatomy of the human prefrontal operculum to the emergence of speech
Source: Commun Biol. 2023 Jul 5;6:693. doi: 10.1038/s42003-023-05066-9 (PMC10322890; doi:10.1038/s42003-023-05066-9)
Supplement: Supplementary file 1 — Supplementary Information [file 42003_2023_5066_MOESM1_ESM.pdf]

Supplemental Data 1

The relevance of the unique anatomy of the human prefrontal operculum to the emergence of speech

Authors:

Céline Amiez, Charles Verstraete, Jérôme Sallet, Fadila Hadj-Bouziane, Suliann Ben Hamed, Adrien Meguerditchian, Emmanuel Procyk, Charles R.E. Wilson, Michael Petrides, Chet C. Sherwood, and William D. Hopkins

Supplemental Data 1.

Figures S1, S2, S3, S4, respectively present the sulcal organization of the region of interest in additional typical human, chimpanzee, baboon, and macaque brains.

Fig S1. Typical examples of human brains

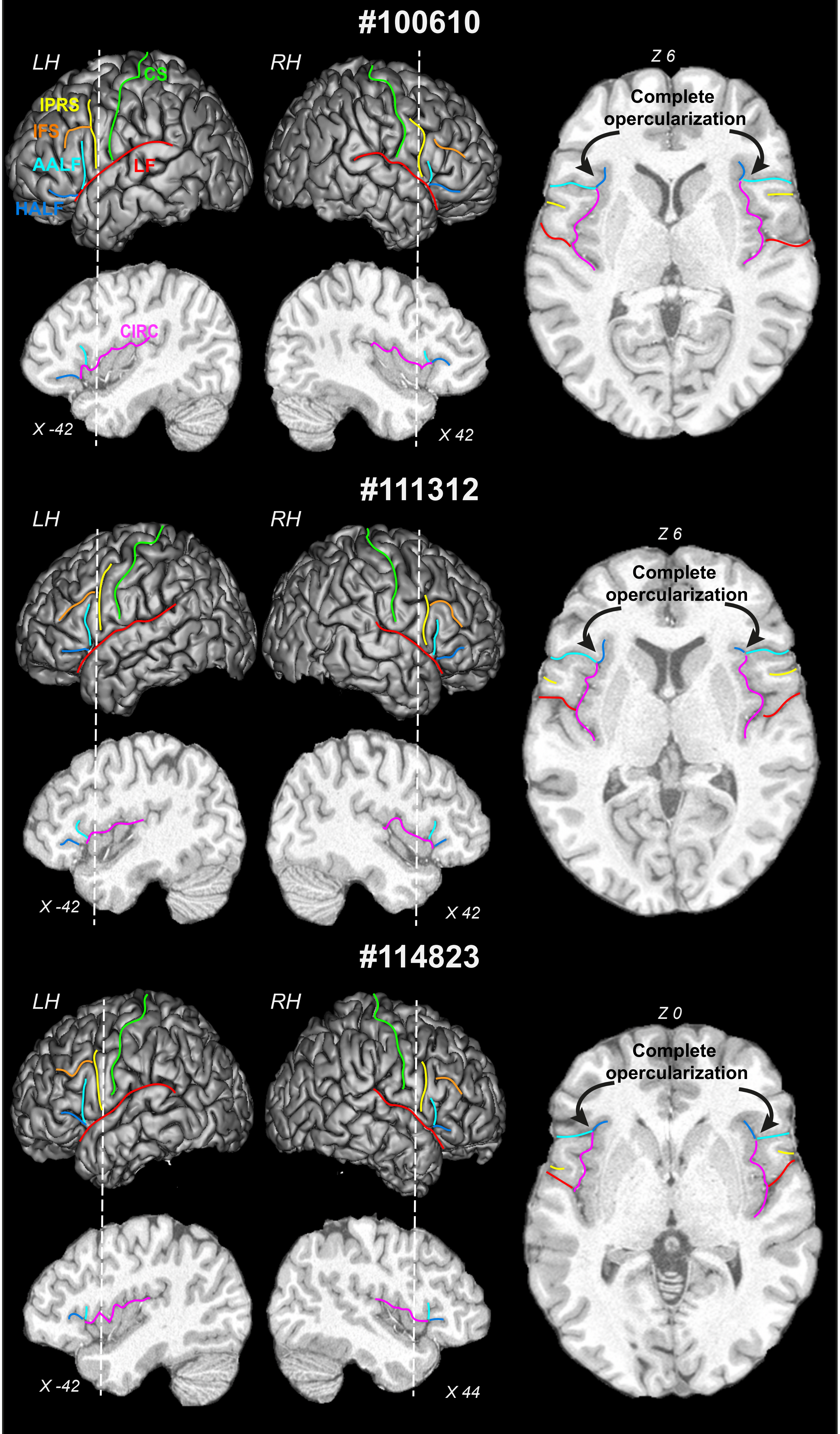

CS, Central Sulcus
LF, Lateral Fissure
IPRS, Inferior Precentral Sulcus
IFS, Inferior Frontal Sulcus
CIRC, CIRCular sulcus
AALF, Ascending Anterior ramus of the Lateral Fissure
HALF, Horizontal Anterior ramus of the Lateral Fissure

Fig S2. Typical examples of chimpanzee brains

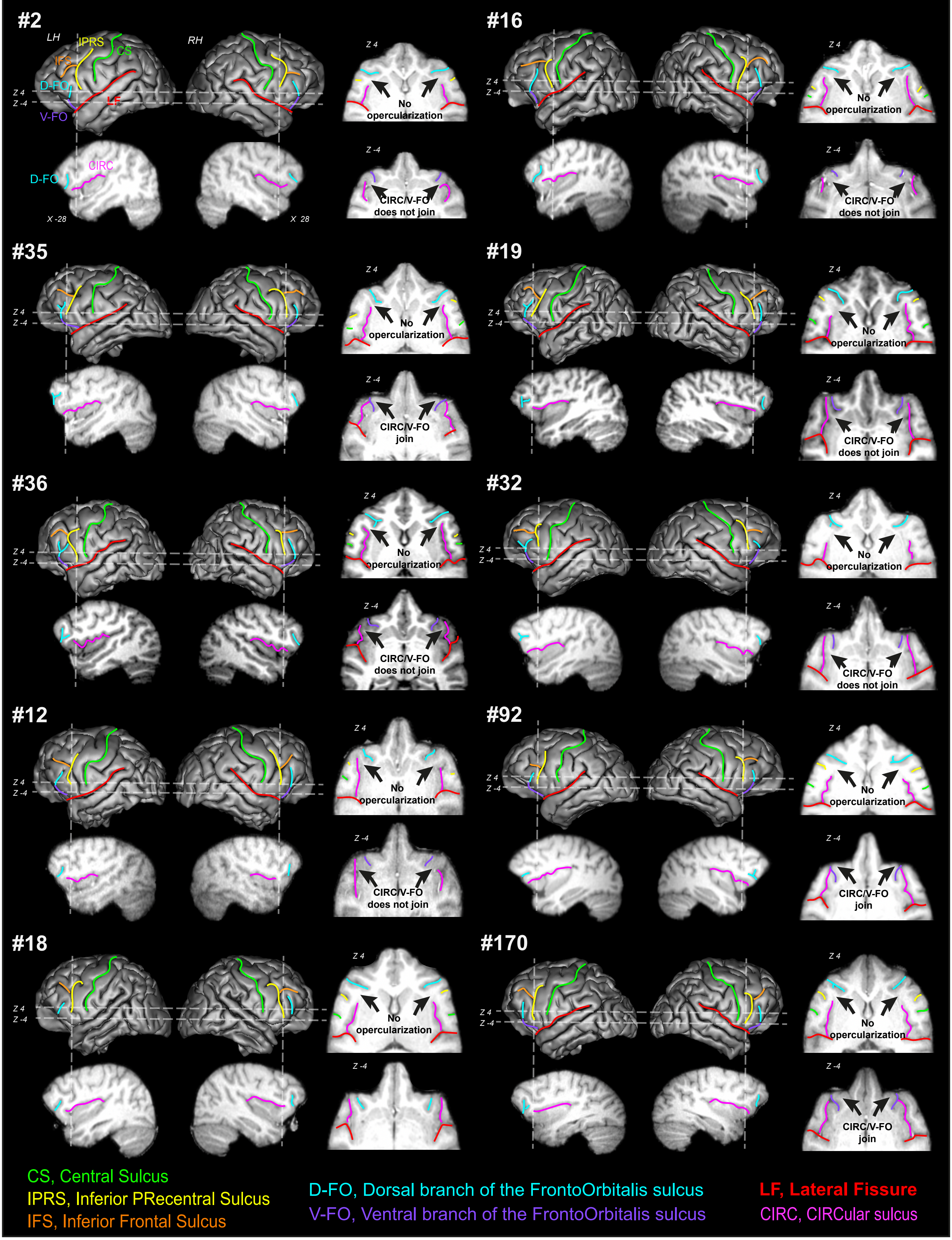

Fig S3. Typical examples of baboon brains

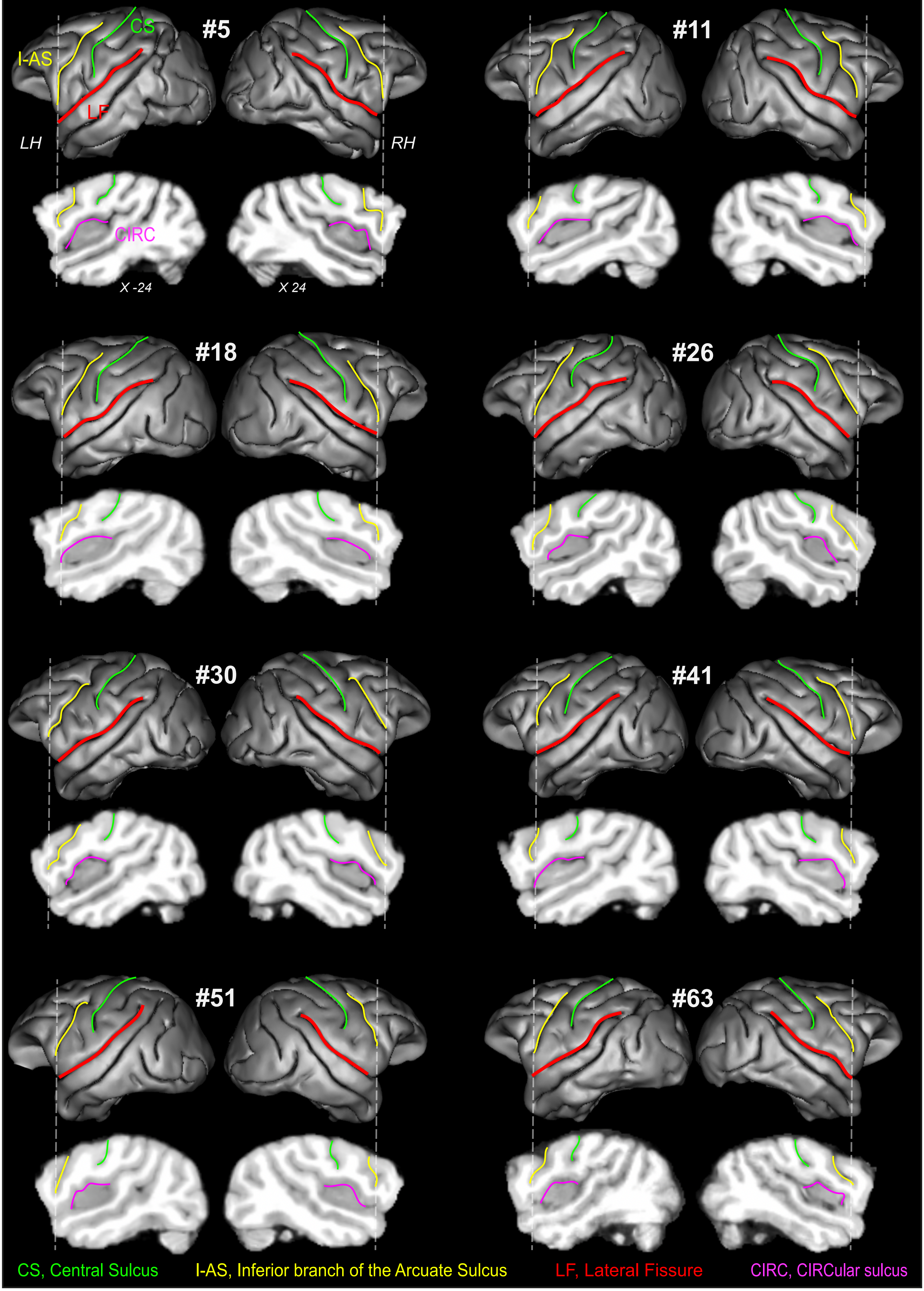

Fig S4. Typical examples of macaque brains

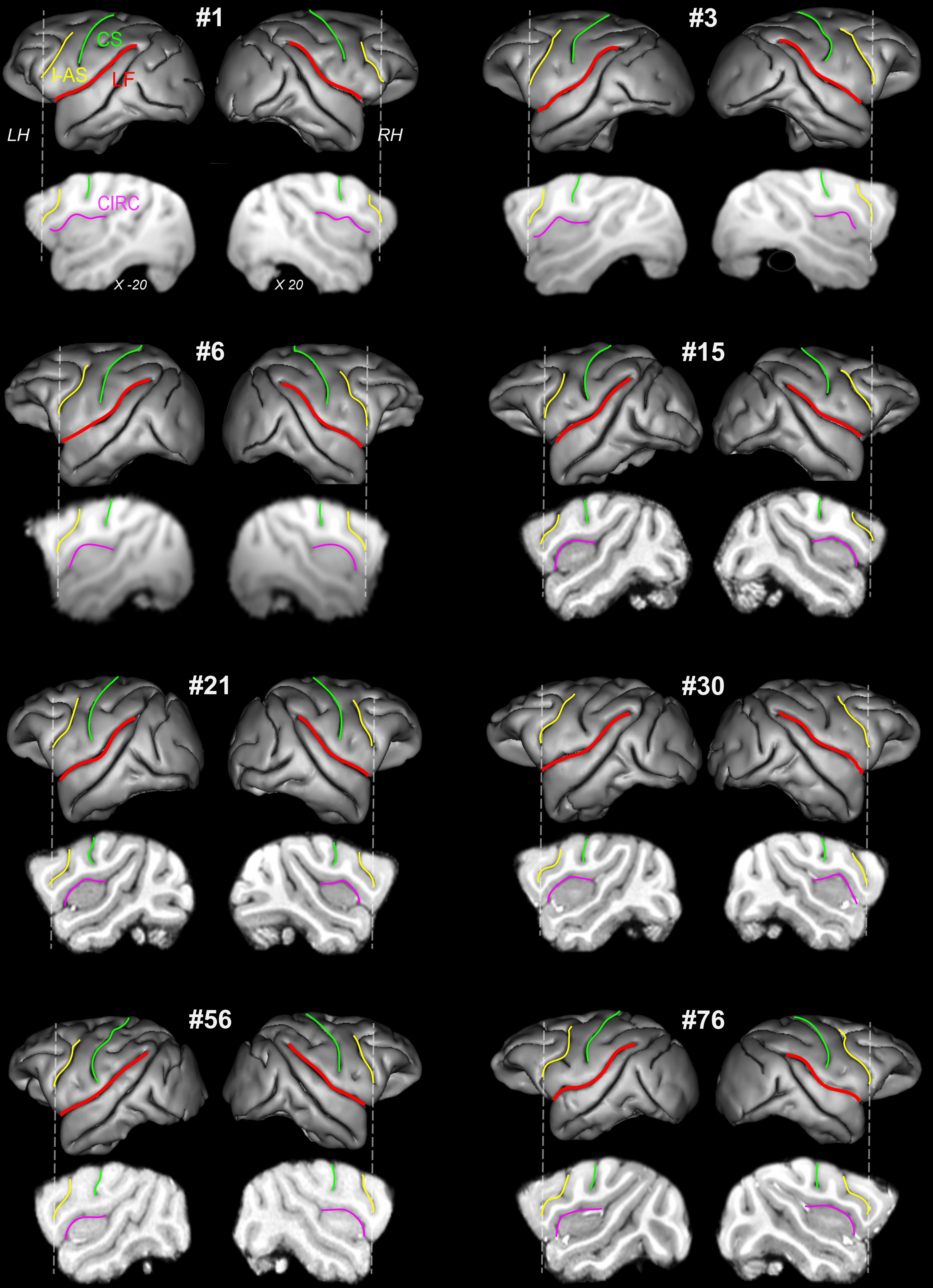

CS, Central Sulcus I-AS, Inferior branch of the Arcuate Sulcus LF, Lateral Fissure CIRC, CIRCular sulcus
