# Supplementary material for: The relevance of the unique anatomy of the human prefrontal operculum to the emergence of speech
Source: Commun Biol. 2023 Jul 5;6:693. doi: 10.1038/s42003-023-05066-9 (PMC10322890; doi:10.1038/s42003-023-05066-9)
Supplement: Supplementary file 2 — Description of Additional Supplementary Files [file 42003_2023_5066_MOESM2_ESM.pdf]

Description of Additional Supplementary Files

File name: Supplemental Data 1
Description: The source data underlying Figures 2-5.
